# Healing of Bone Defects in Pig's Femur Using Mesenchymal Cells Originated from the Sinus Membrane with Different Scaffolds

**DOI:** 10.1155/2019/4185942

**Published:** 2019-09-30

**Authors:** Rita Bou Assaf, Kazem Zibara, Mohammad Fayyad-Kazan, Fatima Al-Nemer, Manal Cordahi, Saad Khairallah, Bassam Badran, Antoine Berbéri

**Affiliations:** ^1^Department of Oral and Maxillofacial Surgery, Faculty of Dentistry, Lebanese University, Beirut, Lebanon; ^2^Laboratory of Cancer Biology and Molecular Immunology, Faculty of Sciences-I, Lebanese University, Beirut, Lebanon; ^3^PRASE, Biology Department, Faculty of Sciences-I, Lebanese University, Beirut, Lebanon; ^4^Department of Histopathology, Faculty of Medicine, Lebanese University, Beirut, Lebanon

## Abstract

**Objective:**

Repairing bone defects, especially in older individuals with limited regenerative capacity, is still a big challenge. The use of biomimetic materials that can enhance the restoration of bone structure represents a promising clinical approach. In this study, we evaluated ectopic bone formation after the transplantation of human maxillary Schneiderian sinus membrane- (hMSSM-) derived cells embedded within various scaffolds in the femur of pigs.

**Methods:**

The scaffolds used were collagen, gelatin, and hydroxyapatite/tricalcium phosphate (HA/*β*TCP) where fibrin/thrombin was used as a control. Histological analysis was performed for the new bone formation. Quantitative real-time PCR (qRT-PCR) and immunohistochemistry (IHC) were used to assess mRNA and protein levels of specific osteoblastic markers, respectively.

**Results:**

Histological analysis showed that the three scaffolds we used can support new bone formation with a more pronounced effect observed in the case of the gelatin scaffold. In addition, mRNA levels of the different tested osteoblastic markers Runt-Related Transcription Factor 2 (RUNX-2), osteonectin (ON), osteocalcin (OCN), osteopontin (OPN), alkaline phosphatase (ALP), and type 1 collagen (COL1) were higher, after 2 and 4 weeks, in cell-embedded scaffolds than in control cells seeded within the fibrin/thrombin scaffold. Moreover, there was a very clear and differential expression of RUNX-2, OCN, and vimentin in osteocytes, osteoblasts, hMSSM-derived cells, and bone matrix. Interestingly, the osteogenic markers were more abundant, at both time points, in cell-embedded gelatin scaffold than in other scaffolds (collagen, HA/*β*TCP, fibrin/thrombin).

**Conclusions:**

These results hold promise for the development of successful bone regeneration techniques using different scaffolds embedded with hMSSM-derived cells. This trial is registered with NCT02676921.

## 1. Introduction

Bone defects due to traumatic injury or surgical excision of infected, neoplastic, or malformed bone tissue may not heal spontaneously, especially in the elderly. Nowadays, clinical procedures for bone repair include different tissue graft strategies for restoring the anatomical and functional status of the bone. For instance, autografts, being nonimmunogenic and with high osteogenic potential, represent the gold standard for bone tissue regeneration [1]. However, several complications such as pain, pathogenic infection, bleeding, and scarring at the donor site can limit their usage [2]. Alternatively, allografts have been used, but they raise critical issues due to their low osteogenic potential, risk of infection, and immunogenic rejection [3]. Hence, developing clinical alternatives has been a long-standing objective. During the past decade, bone tissue engineering, using bone graft substitutes, has emerged as a promising innovative therapeutic approach for bone repair and regeneration [4]. The concept of bone tissue engineering is based on the design of novel biomaterials that have the capacity of mimicking native bone behavior in terms of both mechanical and osteogenic properties [5]. Engineering of bone regeneration *in vitro* relies on the use of osteoprogenitor cells, biomaterial scaffold, growth factors, and an appropriate culture environment [6]. Osteoprogenitor cells are preferably isolated from the recipient, then expanded in culture, and seeded on a scaffold that is gradually degraded as osteogenic differentiation proceeds. These cells are then either cultivated *in vitro* to generate an engineered graft or implanted directly *in vivo* to stimulate bone regeneration [7]. Among the available osteoprogenitor cells, mesenchymal stem cells (MSCs), mainly those derived from the bone marrow (BM) and adipose tissue, have been characterized by a high proliferation capacity and multilineage differentiation potential *in vitro* [8–15]. Human maxillary Schneiderian sinus membrane (hMSSM) was described to contain progenitor cells with similar morphological characteristics and immunological profile characteristics of MSCs [16, 17]. Interestingly, these hMSSM-derived cells showed significant potential to differentiate into cells of osteogenic lineage, thus representing a promising clinical tool for improving implant-based therapies [17]. There has been extensive interest in the use of MSCs in maxillary sinus augmentation (MSA). Recently, a meta-analysis [18] addressed this relatively novel topic by searching MEDLINE, Embase, and Scopus. The authors showed the effectiveness of MSCs in MSA with various scaffold materials in nine studies (seven animals and two human studies). Indeed, a positive effect of stem cells on bone regeneration was found highlighting the potential for cell-based approaches in MSA. The finding that adult MSCs can be operated in vitro, and subsequently form bone in vivo, postulates new therapeutic strategies for regeneration in dentistry [19].

On the other hand, the potential of the scaffold to induce osteogenic cells is highly dependent on its biological and chemical properties and its ability to attach cells and trigger their correct differentiation [5]. A successful scaffold should also be nontoxic, nonimmunogenic, bioactive, biocompatible, biodegradable, and bioresorbable and possess certain mechanical properties. To date, a wide variety of synthetic and natural scaffolds have been applied in regenerative medicine [20]. Among the ones that have been employed in bone tissue engineering are collagen, gelatin, chitosan, hydroxyapatite (HA), tricalcium phosphate (TCP), polycaprolactone (PCL), and poly(lactic-co-glycolic acid) (PLGA) scaffolds [5, 21].

In this study, we have assessed the osteogenic potential of collagen, gelatin, and HA-*β*TCP-fibrin after implantation in pig femur *in vivo*. The implanted scaffolds were either cell-free or charged with hMSSM-derived cells.

## 2. Materials and Methods

### 2.1. Patient Samples

This study was approved by the Institutional Review Board (IRB) of the Lebanese University. hMSSM tissue samples were obtained according to the ethical guidelines after informed consent forms were signed by patients enrolled in the study. A total of 12 hMSSM samples (~2 × 2 cm) were obtained during a surgical nasal approach for treatment of chronic rhinosinusitis, performed under general anesthesia. Smokers and patients with skeletal disorders or systemic diseases were excluded from the study. After the collection, tissue samples were placed in phosphate-buffered saline (PBS) containing 1% penicillin-streptomycin (P/S) at 4°C and processed within 24 hours, as described in our previous study [17].

### 2.2. Isolation and Characterization of hMSSM-Derived Cells

We followed the method described by Berbéri et al. [17]. Briefly, hMSSM samples were extensively washed with PBS supplemented with 1% P/S and cut into small pieces under aseptic conditions. Tissue fragments were incubated with 1 U/ml dispase I solution (Sigma-Aldrich, USA) in PBS at 37°C for 1 hour to separate the epithelial lining from the membrane. Epithelial cells were discarded, and the remaining tissue fragments were treated with 200 collagen digestion units (CDU)/ml of collagenase type II (Sigma-Aldrich, USA) in Hank's balanced salt solution (HBSS) containing 5 Mm calcium chloride at 37°C for 3 hours. Tissues were shook repeatedly during enzymatic incubation. The resulting cells were filtered out with a 40 *μ*m cell strainer (BD Biosciences), and then, hMSSM-derived cells were centrifuged at 900 RPM for 10 minutes.

### 2.3. Culture of hMSSM-Derived Cells in Nonosteogenic Media

We followed the procedure previously described by Berbéri et al. [17]. Isolated cells were plated in T75 cm^2^ with alpha minimum essential medium (*α*-MEM) (Sigma-Aldrich, USA) containing 10% fetal bovine serum (FBS), 1% P/S, and 2 Mm L-glutamine (nonosteogenic media) and cultured in an incubator at 37°C, 5% CO_2_. Daily morphologic characterization was observed with an inverted microscope, and the culture solution was changed two times per week. When the medium was changed, nonadherent cells were removed whereas adherent cells were cultured. When culture dishes became nearly confluent, cells were passaged with trypsin-ethylenediaminetetraacetic acid (EDTA). Cells were assayed at passage 3 for their osteogenic potential.

### 2.4. Preparation of Scaffolds

The procedure used to prepare each of the scaffolds is detailed in our previous report [22].

### 2.5. Animals

The study protocol was reviewed and approved by the ethical committee of the Lebanese University. A total of 12 male Landrace pigs (4 months old) with an average weight of 38 ± 2 kg were included in the study. The femur bone was chosen because of its cortical morphology, as well as its large uniform area, which makes it ideal for multiple defect assessments. The animals were maintained in separate rooms under standard laboratory conditions of water and diet.

### 2.6. Surgical Procedures

**Figure 1:**
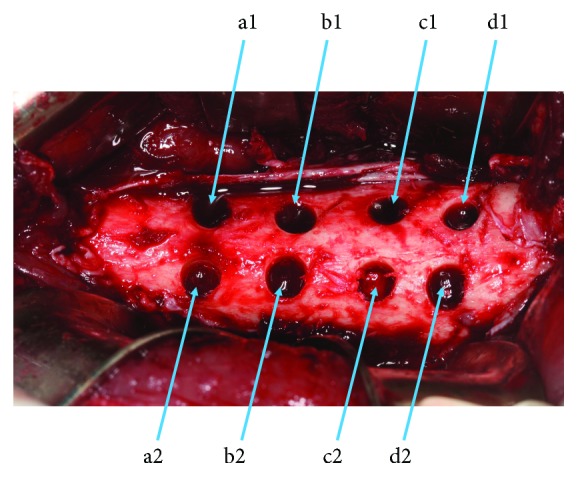
The eight defects were divided into two groups depending on the type of scaffolds and cells that they have received. a1: mesenchymal sinus membrane cell with collagen. b1: mesenchymal sinus membrane cell with gelatin (hemostatic sponge). c1: mesenchymal sinus membrane cell with *β*TCP and HA. d1: mesenchymal sinus membrane cell with fibrin and thrombin. a2: collagen without stem cells. b2: gelatin without stem cells. c2: *β*TCP and HA without stem cells. d2: fibrin and thrombin.

One hour before surgery, the pigs were anesthetized by an intramuscular injection (IM) with a combination of 1 mg/0.45 kg xylazine (AnaSed® 100 mg/ml, Mandeville, Louisiana, USA) and 0.04 mg/1 kg atropine sulfate (SA RX Veterinary Products, Westlake, TX, USA). The surgical sites were then shaved and swabbed with 4% chlorhexidine gluconate surgical scrub (BactoShield® CHG, STERIS Corporation, Road Mentor, USA). The surgery was performed in aseptic conditions and under general anesthesia by an intravenous injection (IV) of 20 mg/kg ketamine hydrochloride (Panpharma, France). A longitudinal skin incision was made on the medial side of the right femur. The subcutaneous tissues and the periosteum were incised in order to expose the bone surface. Eight bone cavities were placed at 5 mm intervals, each of 5 mm in diameter and 6 mm in depth, and were prepared in each animal. These cavities were prepared under saline irrigation (0.9% NaCl) with a bone trephine drill (Salvin Dental Specialties, Inc., USA) at 2000 rpm. The eight defects were divided into two groups depending on the type of scaffolds and cells that they received. A total of 4 different scaffolds were tested in this study: fibrin-thrombin-collagen, fibrin-thrombin-gelatin, fibrin-thrombin-HA-*β*TCP, and fibrin-thrombin alone. The first group of four cavities contained cells along with the 4 different scaffolds (a1: cells+fibrin-thrombin-collagen, b1: cells+fibrin-thrombin-gelatin, c1: cells+fibrin-thrombin-HA-*β*TCP, and d1: cells+fibrin-thrombin alone). The second group of four cavities was used as controls and was filled with the same scaffolds without any cells (a2, b2, c2, and d2, respectively) (Figure 1). All scaffolds were prepared and kept overnight in the incubator, prior to implantation in pigs.

After placing all graft materials, the bone was recovered by a 2 × 8 cm collagen wound dressing (CollaTape, Zimmer Biomet Dental, Palm Beach Gardens, FL, USA). The flap consisting of periosteum and subcutaneous tissue was adjusted and closed by a layer using resorbable interrupted sutures (Vicryl® 0, Ethicon Johnson & Johnson, Somerville, NJ). The skin was sutured with interrupted sutures using nonresorbable monofilament suture (ETHILON® 0, Ethicon Johnson & Johnson, Somerville, NJ). After surgery, the pigs received IM medication treatment; a combination of 200 mg/250 mg penicillin/streptomycin antibiotics (Pen-Strep® 1 ml/25 kg, Norbrook, Farmacy.co. Warnham, West Sussex, UK) every 12 hours for a duration of 5 days and 3 ml/33 kg ketoprofen as anti-inflammatory drug (ketoprofen, Norbrook, Farmacy.co. Warnham, West Sussex, UK) once per day, for 3 days. Body temperature, pulse, and respiration were closely monitored for potential complications. The sutures were removed after 10 days.

### 2.7. Sacrifice

The 12 pigs were divided into 4 groups of 3 pigs each, depending on the time of sacrifice. The first group of 3 pigs, with 6 femurs, was sacrificed at 2 weeks postsurgery (group 1). The second, third, and fourth groups (groups 2, 3, and 4) were sacrificed at 4, 6, and 8 weeks postsurgery, respectively. The pigs were sacrificed using a lethal dose of 150 mg/kg ketamine HCl IV injection (Panpharma, France), and the femur bone was resected. Afterwards, circular blocks encompassing each drill defect were cut and frozen prior to further processing. Rectangular block sections of the femur were removed and fixed in 4% paraformaldehyde (PFA) for histology and immunohistochemistry.

### 2.8. Histology

Bones were fixed in 10% neutral formalin one week before decalcification with 0.5 M EDTA in saline (pH 7.4). Sections were taken from the center of each defect when identified. Whereas when not identified, it was taken from the scar replacing the defect. Samples were then dehydrated using gradual ethanol series and embedded in paraffin. They were cut at 6 *μ*m and stained with hematoxylin and eosin (H&E) staining in order to be examined under light microscopy. H&E staining allowed detection of new bone formation. Bone was presented as a compact structure in a dark red color. Fibroblastic reaction was displayed in a pink color.

### 2.9. Immunohistochemical Staining

Immunohistochemistry was performed on decalcified and paraffin-embedded sections. The latter were dewaxed using EZ Prep, hydrated, and heat-treated for 30 min at 60°C. Sections were then incubated according to the manufacturer's instructions, at room temperature, with the prediluted monoclonal antibodies RUNX-2 (1 : 200, Abcam) and OCN (1 : 200, Abcam) to identify bone formation whereas vimentin (1 : 80, Biogenex) was used to identify mesenchymal cells and osteoblasts (Table 1). The immunohistochemical study was done on an automatic immunostainer (Ventana-Benchmark XT). All slides were visualized using an Olympus BX51 microscope, and images were captured by digital camera and cellSens software. The immunohistochemical expressions of the markers are the following: for the RUNX-2, the nuclei of osteoblasts; for the OCN, the bone matrix, osteocytes, and osteoblasts; and for the vimentin, the osteocytes, osteoblasts, and mesenchymal cells.

### 2.10. Quantitative Real-Time Polymerase Chain Reaction

We followed the method described by Berbéri et al. [17]. Real-time PCR was performed in order to examine the mRNA expression of specific osteoblastic markers such as ALP, RUNX-2, OCN, OPN, ON, and type 1 collagen (COL1). Primers used were the following: ALP, F: GGGGGTGGCCGGAAATACAT and R: GGGGGCCAGACCAAAGATAGAGTT; RUNX-2, F: CCGCACGACAACCGCACCAT and R: CGCTCCGGCCCACAAATCTC; Col1, F: GAGGGCCAAGACGAAGACATC and R: CAGATCACGTCATCGCACAAC; OCN, F: TCACACTCCTCGCCCTATTGG and R: TCACACTCCTCGCCCTATTGG; OPN, F: AGACCCCAAAAGTAAGGAAGAAG and R: GACAACCGTGGGAAAACAAATAAG; and ON, F: CCTGGAGACAAGGTGCTAACAT and R: CGAGTTCTCAGCCTGTGAGA. Briefly, total RNA was isolated using TRIzol reagent (Invitrogen) according to the manufacturer's instructions. First-strand cDNA was synthesized from 1 *μ*g of extracted RNAs using the RevertAid 1st-Strand cDNA Synthesis Kit (Fermentas). After cDNA synthesis, PCR was performed using 1 *μ*g of cDNA mixed with 10 *μ*l SYBR Green and loaded in duplicates with 5 *μ*M forward and reverse primers. PCR cycling conditions were as follows: initial denaturation at 95°C for 10 min, then 45 cycles with denaturation at 95°C for 15 s, annealing temperature for 15 s, and extension at 72°C for 15 s. Basic expression levels for the genes of interest were quantified after normalization to glyceraldehyde-3-phosphate dehydrogenase in human (hGAPDH) mRNA levels, using human specific primers (hGAPDH housekeeping gene set) (Roche Applied Science, Branford, USA).

### 2.11. Statistics

Data are presented as means ± SEM of at least three independent experiments and analyzed using Student's *t*-test. *p* values  < 0.05 (^∗^) and  < 0.01 (^∗∗^) were considered as significant.

## 3. Results

### 3.1. Histological Evaluation of Bone Formation

**Figure 2:**
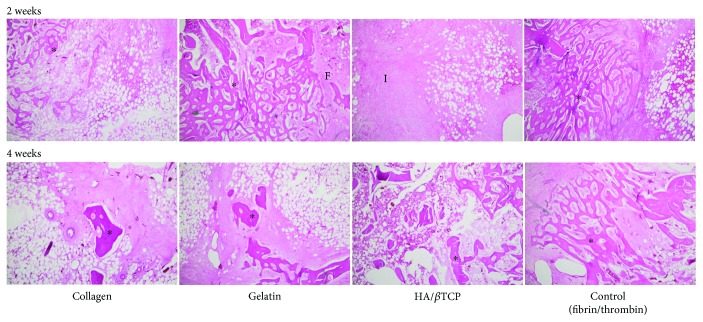
Hematoxylin and eosin staining of histological micrographs from paraffin-embedded scaffolds implanted in the femur of pigs at 2 and 4 weeks. Asterisks (^∗^) represent the new bone formation and F corresponds to the fibroblastic reaction while I represents the inflammatory reactions. New bone formation was detected in groups implanted with scaffolds along with the hMSSM cells, in comparison with the control group with scaffolds alone. Magnification is 40x.

The host's response to the scaffolds, with or without cells, after 2 and 4 weeks of implantation was first determined. After 2 and 4 weeks, new bone formation was detected in the center of the bone cavity of the first group (*n* = 3 pigs) implanted with cell-embedded scaffolds, in comparison with those with scaffolds alone (control group) (Figure 2). Bone formation, represented by the dark red structures, appeared to be more prominent in the second group (4 weeks), compared to the first group (2 weeks). It is important to note that bone formation was more evident and obvious in the gelatin group in comparison with collagen, HA/*β*TCP, or fibrin/thrombin control group (Figure 2). A fibroblastic and inflammatory reaction was observed in all groups.

**Figure 3:**
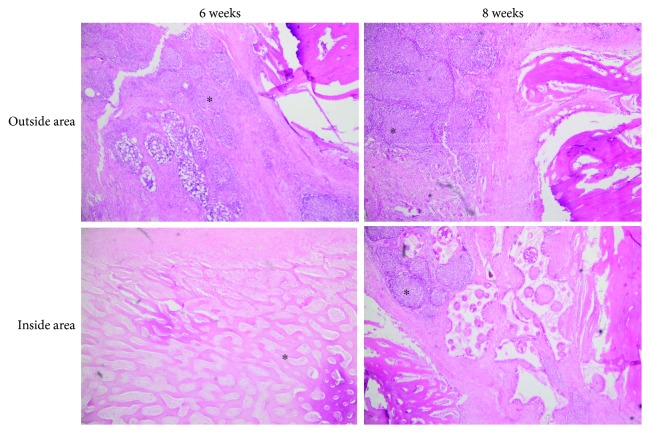
Hematoxylin and eosin staining of histological micrographs from paraffin-embedded scaffolds implanted in the femur of pigs at 6 and 8 weeks. Asterisks (^∗^) represent the mature bone in the inner and outer areas of the scaffolds. Note the presence of a chronic inflammatory exudate within the sections. Magnification is 40x.

The amount of bone-like tissue clearly increased after 4 weeks. Well-formed bone spicules were visible, mainly in the gelatin group. After six and eight weeks (groups 3 and 4), all defect cavities were filled with bone. New bone formation could not be detected anymore. Interestingly, hematoxylin and eosin staining demonstrated the presence of mature bone formation in the inner and outer areas of the scaffolds, especially in groups 3 and 4, after 6 and 8 weeks (Figure 3).

### 3.2. Expression Levels of Osteogenic Markers in Cell-Embedded Scaffolds

The ability of the different tested scaffolds to induce osteogenic differentiation of hMSSM-derived cells was assessed by two different techniques: quantitative real-time PCR (qRT-PCR) and immunohistochemistry (IHC).

In a first step, qRT-PCR analysis was performed on hMSSM-derived cells being seeded within the different tested scaffolds and isolated from the different implants after 2 and 4 weeks. It is important to note that since the defect cavities were completely filled after 6 and 8 weeks and since we could not observe any histological difference between them, these 2 time points were excluded from all latter experiments. Transcription levels of different osteoblastic markers (RUNX-2, ON, OCN, OPN, ALP, and COL1) were examined.

**Figure 4:**
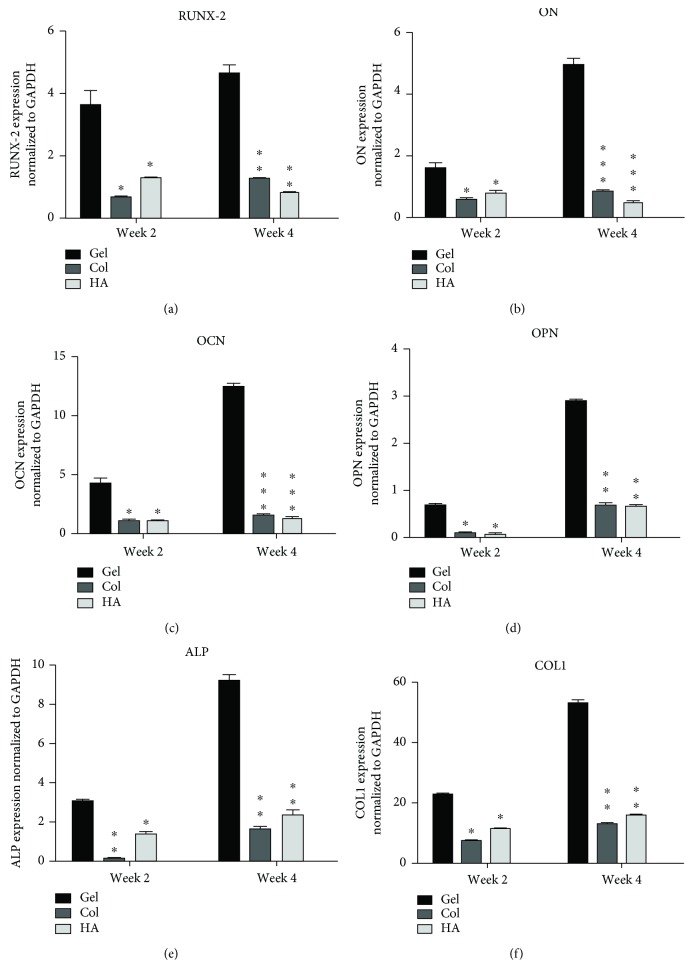
Quantitative real-time PCR (qRT-PCR) of different osteoblastic markers. (a) RUNX-2, (b) ON, (c) OCN, (d) OPN, (e) ALP, and (f) COL1 mRNA levels in cell-embedded scaffolds from different implants at 2 or 4 weeks. The expression levels are relative to those obtained in cells+fibrin-thrombin (control). Data were normalized to GAPDH levels. Each value represents a mean ± SEM for three independent experiments (*n* = 3). ^∗^*p* < 0.05, ^∗∗^*p* < 0.01, and ^∗∗∗^*p* < 0.001*vs.* cells with gelatin scaffold (Student's *t*-test).

The results showed that groups implanted with cell-embedded scaffolds (collagen, gelatin, HA/*β*TCP) along with cells demonstrated significantly higher mRNA levels for all tested genes and for the 2 time points (2 weeks and 4 weeks), in comparison with cell-embedded control scaffold (fibrin/thrombin) and with cells seeded on collagen or HA/*β*TCP (Figure 4).

Interestingly, mRNA expression levels for RUNX-2, ON, and OPN, at 4 weeks, were ~3- to 5-fold significantly higher in cell-embedded gelatin scaffolds than in cells seeded on collagen or HA/*β*TCP scaffolds (Figure 4).

Moreover, OCN, ALP, and COL1 mRNA levels increased further and were ~10 (in case of OCN and ALP)- and ~50 (in case of COL1)-fold higher in cell-embedded gelatin scaffolds.

**Figure 5:**
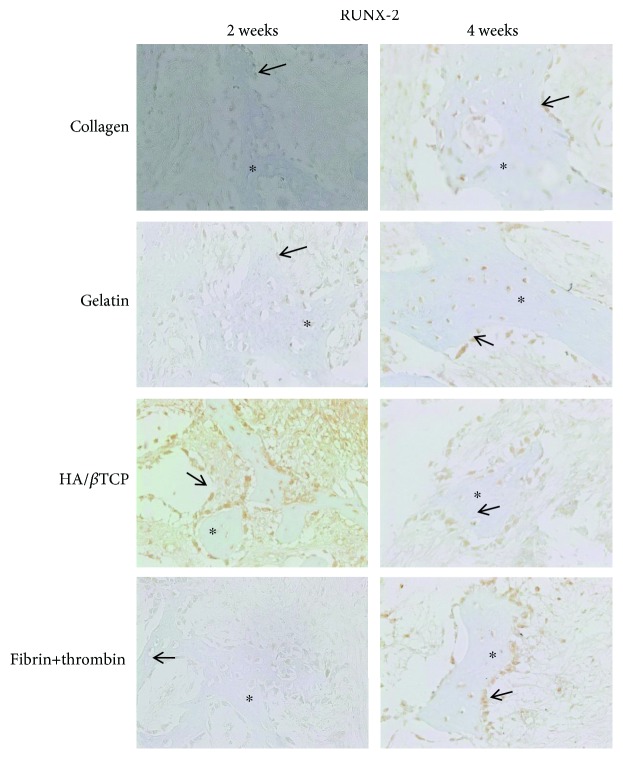
Immunohistochemistry (IHC) of RUNX-2 osteoblastic marker. Expression of RUNX-2 protein in scaffold-embedded hMSSM-derived cells from different implants after 2 or 4 weeks. Asterisks (^∗^) represent the new bone formation while solid arrows (→) correspond to the positively stained osteoblast cells.

**Figure 6:**
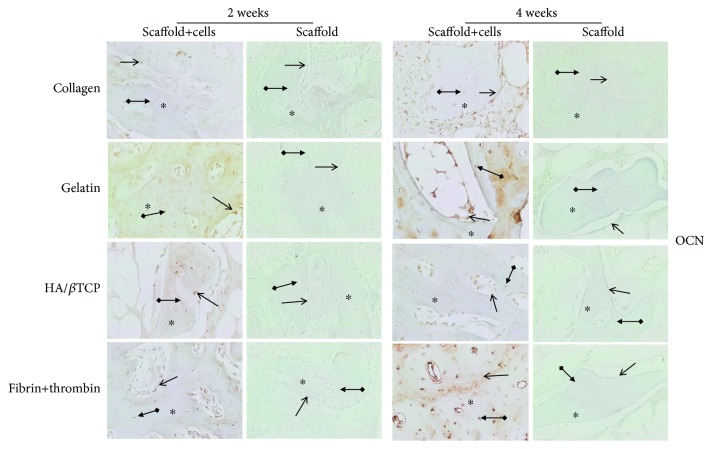
Immunohistochemistry (IHC) of OCN osteoblastic marker. Expression of OCN protein in scaffold-embedded hMSSM-derived cells from different implants after 2 or 4 weeks. Staining showed a very clear differential expression of OCN in osteocytes, osteoblasts, hMSSM-derived cells, and bone matrix. Interestingly, OCN was more abundant, at both time points, in gelatin scaffold-embedded cells than the other scaffolds (collagen, HA/*β*TCP, and fibrin/thrombin). Asterisks (^∗^) represent the new bone matrix formation and regular arrows (→) correspond to the positively stained osteoblast cells while bold arrows (•→) correspond to the positively stained osteocytes.

**Figure 7:**
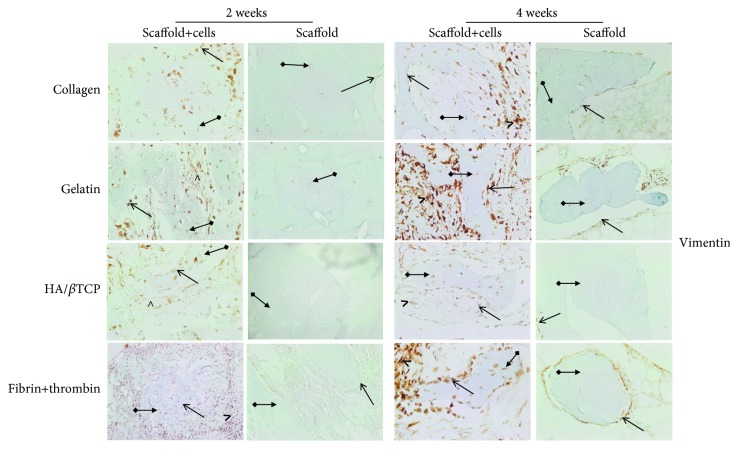
Immunohistochemistry (IHC) of vimentin osteoblastic marker. Expression of vimentin protein in scaffold-embedded hMSSM-derived cells from different implants after 2 or 4 weeks. Staining showed a very clear differential expression of vimentin in osteocytes, osteoblasts, hMSSM-derived cells, and bone matrix. Interestingly, vimentin was more abundant, at both time points, in gelatin scaffold-embedded cells than the other scaffolds (collagen, HA/*β*TCP, and fibrin/thrombin). Asterisks (^∗^) represent the new bone matrix formation and solid short arrows correspond to the positively stained osteoblast cells while solid long arrows correspond to the positively stained osteocytes.

In a second step, IHC was used to assess the expression of RUNX-2, OCN, and vimentin osteogenic markers at the protein level. RUNX-2 (Figure 5), OCN (Figure 6), and vimentin (Figure 7) were clearly detected at both time points (2 and 4 weeks), in scaffold-embedded cells, but not in control cells. Indeed, RUNX-2 was detected in the nuclei of osteoblasts at 2 and 4 week time points (Figure 5). On the other hand, there was a very clear and differential expression of OCN (Figure 6) and vimentin (Figure 7) in osteocytes, osteoblasts, hMSSM-derived cells, and bone matrix. Interestingly, these osteogenic proteins were more abundant, at both time points, in cell-embedded gelatin scaffolds than in the other scaffolds (collagen, HA/*β*TCP, and fibrin/thrombin).

## 4. Discussion

In the present study, we evaluated the capacity of different biomaterials to induce ectopic bone formation *in vivo*, after their transplantation in the femur of pigs either as cell-free scaffolds or as scaffold-embedded hMSSM-derived cells. In the healing period, all pigs remained healthy during the study period and showed no signs of complication or side effects. Our histological evaluation, by qRT-PCR and IHC analysis, clearly demonstrated new bone formation triggered by the different scaffolds, with varied potentials depending on the properties of the material used. Our data revealed that bone formation was more prominent in pigs transplanted with hMSSM-derived cells embedded in gelatin scaffold compared to collagen, HA/*β*TCP, or control fibrin/thrombin scaffolds. This is consistent with our previous *ex vivo* study showing that gelatin scaffold showed higher osteoinductive potential than collagen, HA/*β*TCP, or control fibrin/thrombin scaffolds [22]. This varied osteoinductive potential could be attributed to the distinct physical, chemical, and biological properties of the tested scaffolds [21, 22]. For instance, despite the ability of collagen scaffolds to enhance osteoblastic differentiation and function *in vitro*, the application of these scaffolds is limited by their rapid degradation [23, 24]. Moreover, the poor mechanical properties of collagen scaffolds render them unsuitable to be applied in load-bearing sites [23]. On the other hand, despite the ability of HA/*β*TCP scaffolds to induce osteogenic differentiation, it is well described that cell survival, proliferation, and differentiation supported by HA/*β*TCP vary depending on the HA/*β*TCP ratio [7]. Further, gelatin sponges, being characterized by their structural stability including slow biodegradation, biocompatibility, and capacity to support osteogenic differentiation [25], have been demonstrated as a suitable implant for bone regeneration, thus useful for repair of bone defects [26–28]. Previous reports have proved the ability of scaffolds embedded with hMSSM-derived cells to induce new bone formation *in vivo.* For instance, it has been demonstrated that HA/*β*TCP scaffolds embedded with hMSSM-derived cells can generate new bone formation in a mouse model, mainly in the case of OroGraft and ProOsteon [29]. In our study, we used the pig to study human bone regeneration. Pigs have a bone anatomy and morphology similar to humans as well as conserved bone healing and remodeling mechanisms, which makes them an ideal model system [30]. In addition, pigs have been successfully used in multiple bone studies involving bone fracture, osteonecrosis of femoral head, face reconstruction, and others [31].

## 5. Conclusion

The present study demonstrates the ability of different scaffolds, mainly gelatin, embedded with hMSSM-derived cells to induce bone formation in pigs. In clinical practice, and during sinus lifting surgery, absorbable collagen sponges can provide a matrix for tissue ingrowth: blood platelets are first attracted, then aggregate on the collagen molecules, and then release coagulation factors that work with plasma factors to initiate bone formation. Gelatin scaffolds could therefore hold promise for bone repair and regeneration especially in individuals with reduced regenerative potential.

## Figures and Tables

**Table 1:** Immunohistochemistry reagent.

Primary antibody	Clonality	Dilution	Incubation period	Target	Cellular localization
RUNX-2	Polyclonal	1 : 200	32 min	Osteoblasts	Nuclear
Osteocalcin	Monoclonal	1 : 200	32 min	Osteoblasts, osteocytes, and bone matrix	Nuclear and cytoplasmic
Vimentin	Monoclonal	1 : 80	32 min	Mesenchymal cells including osteocytes and osteoblasts	Cytoplasmic

## Data Availability

The data used to support the results, the analysis, and the findings of this study are included within the article.
